# Attention Gates the Selective Encoding of Duration

**DOI:** 10.1038/s41598-018-20850-y

**Published:** 2018-02-06

**Authors:** Jim Maarseveen, Hinze Hogendoorn, Frans A. J. Verstraten, Chris L. E. Paffen

**Affiliations:** 1 Department of Experimental Psychology, Helmholtz Institute, Utrecht University, Utrecht, The Netherlands; 2 School of Psychology, The University of Sydney, Sydney, NSW 2006 Australia; 3 Melbourne School of Psychological Sciences, The University of Melbourne, Victoria, 3010 Australia

## Abstract

The abundance of temporal information in our environment calls for the effective selection and utilization of temporal information that is relevant for our behavior. Here we investigated whether visual attention gates the selective encoding of relevant duration information when multiple sources of duration information are present. We probed the encoding of duration by using a duration-adaptation paradigm. Participants adapted to two concurrently presented streams of stimuli with different durations, while detecting oddballs in one of the streams. We measured the resulting duration after-effect (DAE) and found that the DAE reflects stronger relative adaptation to attended durations, compared to unattended durations. Additionally, we demonstrate that unattended durations do not contribute to the measured DAE. These results suggest that attention plays a crucial role in the selective encoding of duration: attended durations are encoded, while encoding of unattended durations is either weak or absent.

## Introduction

Temporal information is crucial to our interaction with the external world. Analyzing the temporal order and duration of events allows us to learn about the temporal regularities in the world around us. We can use this knowledge to predict future events, guide our decisions, and plan the timing of our actions^1,2^. Most visual scenes contain numerous sources of temporal information. In a scene containing multiple events, each event contains information about its own duration as well as information about the time between different events. As a result, the amount of temporal information available in our environment can become very large. Despite this abundance of temporal information, most temporal information is not relevant for our immediate behavior. This creates the need for effective selection of relevant temporal information, to avoid irrelevant information from affecting our behavior.

Visual attention provides a means by which relevant visual information can be selected against concurrent, irrelevant information^3^. For example, directing attention towards one of multiple objects or features during adaptation leads to modulation of the resulting after-effects for these objects or features^4–9^. These modulations of after-effect magnitude demonstrate differential processing as a result of attentional selection, and have been proposed to reflect changes in the strength of encoding of attended versus unattended information^4^. This proposal is supported by neurophysiological studies demonstrating that attention modulates the response of visual neurons by increasing both response amplitude and selectivity^10–14^. Together, these results suggest that visual attention provides a mechanism for the selective encoding of relevant versus non-relevant stimulus information by modulating the degree to which visual information is encoded.

While the role of attention in the selection of non-temporal properties is well established, there has been little investigation into its role in the selection of temporal properties. Several studies have demonstrated that the extent to which an event is attended can influence the perceived duration of that event^15–17^. However, these studies do not address the role of attention in situations where multiple sources of duration information are present. This is surprising, as several theories of duration encoding have stressed the role of attention in gating duration information for subsequent encoding^18–22^. In the current study, we investigated the role of attention in the selective encoding of duration. We presented participants with multiple duration signals and investigated whether attending one source of duration information modulated duration encoding. We used duration-adaptation^23^ to probe duration encoding and measured whether allocating attention towards one of multiple sources of duration information modulated the resulting duration after-effect (DAE). The DAE is a repulsive after-effect in which adaptation to a specific duration in one modality leads to a repulsive shift in the perceived duration of post-adaptation stimuli presented in the same modality^23–26^. For example, adaptation to an 800 ms visual stimulus will lead to subsequent presentations of a visual stimulus with a shorter duration (i.e. 400 ms) to be perceived as even shorter, and presentations of a visual stimulus with a longer duration (i.e. 1200 ms) as even longer. This after-effect of adapting to duration is interpreted to reflect selective adaptation of duration-tuned channels as a result of the repeated encoding of their preferred duration^23^. In line with this interpretation, modulation of the DAE is taken to reflect changes in the strength of encoding of the presented duration information.

**Figure 1:**
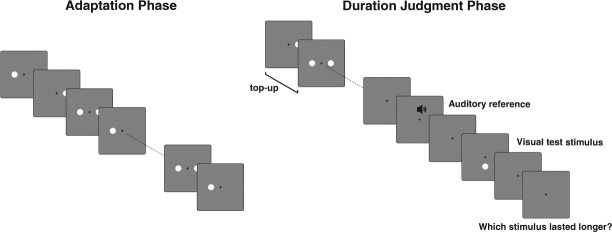
A schematic depiction of the experimental procedure. Adaptation Phase (left): Participants adapted to two asynchronous streams of duration stimuli presented left and right of fixation. Each stream consisted of repetitions of a single duration (i.e. 200 ms on the left, 800 ms on the right). In each stream ~10% of presentations consisted of duration oddballs with a shorter or longer duration. The participants’ task was to maintain fixation and detect duration oddballs on one side (left or right) for the full duration of the adaptation phase. Duration Judgment Phase (right): Participants completed a duration judgment task in which they compared the duration of an auditory reference to that of a visual test stimulus. To maintain adaptation, a short top-up oddball detection phase preceded the duration judgment.

In Experiment 1, participants adapted to two asynchronous streams of stimuli, each consisting of repetitions of a single duration stimulus that lasted either 200 or 800 ms (Fig. 1). To probe the modulatory effect of attention, participants were instructed to detect duration oddballs in either the 200 or the 800 ms stream. Following adaptation, we measured the resulting DAE using a duration judgment task, in which participants compared the duration of an auditory reference (400 ms) to that of a visual test stimulus. We predicted that if attention modulates duration encoding, adaptation to the attended duration should increase relative to the adaptation to the unattended duration. In other words, attending the 800 ms durations should lead to a 400 ms test stimulus being perceived as shorter, compared to attending the 200 ms durations (implying stronger encoding of the attended duration). In a second experiment, we aimed to quantify the contribution of attended versus unattended durations to the DAE. To this end, we utilized adaptation to a reference duration – which predicts no DAE – to establish a baseline against which to measure the contribution of the attended and unattended durations. By doing so, we were able to probe the extent to which attended and unattended duration are encoded for further processing.

## Results

### Experiment 1

**Figure 2:**
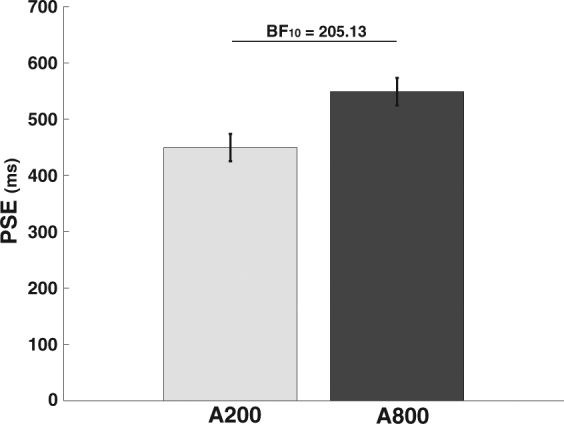
Average Point of Subjective Equality (PSE) for the cross-modal duration judgments following adaptation. Larger PSE values reflect shorter perceived duration for the test stimuli. Error bars reflect within-subject standard error^46,47^. Bayes factors were used to describe the evidence for the alternative hypothesis that attention modulates the DAE. BF_10_ > 3.0 is considered evidence for the H_a_^50,51^.

To examine the modulatory effect of attention on the DAE, we calculated the average Point of Subjective Equality (PSE) for when participants were attending either the 200 ms stream (A200) or the 800 ms stream (A800). Average PSEs for each attention condition can be found in Fig. 2. We found clear evidence for attentional modulation of the DAE: attending a stream of 200 ms stimuli lead to a longer perceived duration of subsequent test stimuli (M_PSE_ = 449.5, SD = 108.3) compared to attending a stream of 800 ms stimuli (M_PSE_ = 548.9, SD = 124.1; Bayesian t-test: BF_10_ = 205.34). The Bayes factor (BF) reported here indicates that the current data were 205.34 times more likely to occur under the alternative hypothesis that attention modulated the DAE then under the null hypothesis that no attentional modulation occurred.

This result demonstrates that attention allows for the selective encoding of duration information. This finding that attention modulates the DAE is in line with effects of attentional selection on adaptation for non-temporal feature information^4–7^. Interestingly, the magnitude of attentional modulation is similar in magnitude to the after-effects obtained by studies using adaptation to only a single stream of durations^24^. This suggests that the modulatory strength of attention is relatively large and that unattended durations might not contribute to the measured DAE. In other words, the magnitude of attentional modulation found here could reflect an absence of the encoding of the unattended durations. This possibility was evaluated in Experiment 2.

### Experiment 2

In Experiment 2, we compared the contribution of attended and unattended durations to the measured DAE. Participants performed the oddball detection task under three conditions: repetitions of blobs lasting 200 and 400 ms, while performing the oddball task on the 200 ms blobs (A200|U400) or the 400 ms blobs (A400|U200), or to repetitions of blobs that both lasted 400 ms, while performing the oddball task on one of the 400 ms streams (A400|U400). By using adaptation to the baseline duration (400 ms) – which does not predict a DAE – we could examine the contribution of the attended (A200|U400 vs. A400|U400) and the unattended (A400|U200 vs. A400|U400) durations to the measured DAE.

**Figure 3:**
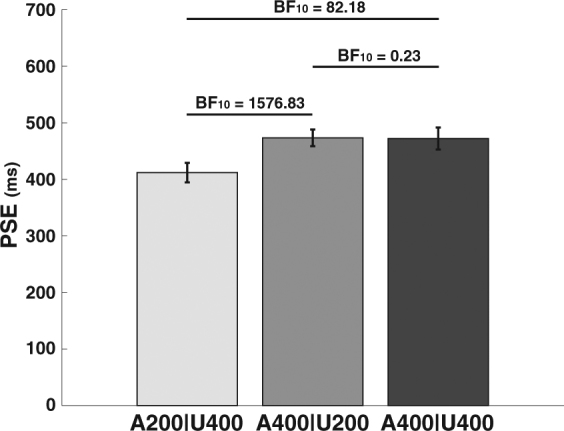
Average Point of Subjective Equality (PSE) for the cross-modal duration judgments following adaptation. Larger PSE values reflect shorter perceived duration for the test stimuli. Error bars reflect within-subject standard error^46,47^. Bayes factors (BF_10_) were used to describe the relative evidence for H_a_ vs. H_0_. BF_10_ larger then 3.0 are considered evidence for the H_a_, while BF_10_ smaller then 1/3 is considered evidence for the H_0_^50,51^.

As in Experiment 1, the data showed that attention modulated the magnitude of the DAE (Fig. 3). A Bayesian within-subject ANOVA revealed evidence for the inclusion of the factor Attention (BF_10_ = 1689.81) in explaining the collected data. To further interpret this result, we conducted separate Bayesian t-tests for each pairwise comparison between the different attention conditions. This analysis showed that participants perceived the test stimuli as having a longer duration in the A200|U400 condition (M = 411.9 SD = 107.6) compared to the A400|U200 (M = 473.3, SD 106.1; BF_10_ = 1576.83) and the A400|U400 conditions (M = 472.1 SD = 120.8; BF_10_ = 82.18) (Fig. 3). These findings corroborate our result from Experiment 1, demonstrating that attention can lead to relative shifts in the encoding of attended versus unattended durations. More importantly, we found evidence that there was no difference in the DAE between the A400|U200 and the A400|U400 conditions (BF_10_ = 0.233: data 4.29 times more likely under H_0_). These results show that the DAE results from adaptation to the attended durations, with no evidence of a contribution of the unattended durations.

## Discussion

In this study, we investigated whether attention gates the encoding of duration. We adapted participants to streams of differing durations and measured whether attending one of those streams modulated the DAE. The results provide strong evidence for attentional modulation of the DAE: test stimuli were perceived as having a longer duration when a stream of shorter durations was attended, compared to when a stream of longer durations was attended. We also investigated the degree to which attended and unattended durations are encoded by probing the contribution of attended and unattended durations to the DAE. We used adaptation to the reference duration (400 ms) – which predicts no DAE – to establish a baseline. We then used this baseline to compare adaptation to a non- reference duration when it was attended (A200|U400 vs. A400|U400) versus when it was not (A400|U200 vs. A400|U400). We found that the attended duration contributed to the DAE while the unattended duration did not. Given that the DAE is thought to result from repeated encoding of duration information, the lack of contribution of unattended durations to the DAE suggests that little to no encoding of the unattended duration occurred.

Earlier work on duration processing has demonstrated that manipulating the extent to which a stimulus is attended can influence the perceived duration of that stimulus^15–17,27,28^. For example, increasing the attentional load during tasks involving duration leads to a decrease in the perceived duration of stimuli^17^. Furthermore, shifting spatial attention when encoding the duration of an event can lead to a decrease in its perceived duration^29^. In line with these findings, several theories of duration processing have included an attentional switch component as part of their model, which can lead to a decrease or increase in perceived duration depending on the state of the switch^18,22^. This switch is often postulated to be a gating mechanism for duration signals, where changes in attention will change the amount of temporal information that is accumulated during an interval. While these studies provide evidence for a link between attention and perceived duration, they focus on manipulating the extent to which a single, task-relevant stimulus is attended. As such, these studies do not provide insight into the selective encoding of a relevant duration that is embedded in a scene with multiple sources of duration information. Our findings clearly demonstrate that attention does not only change the encoding of a single duration, but also plays a crucial role in selecting which of multiple source of duration information are encoded for further processing.

The current study shows that attention allows for the selective encoding of duration information. Given the role of attention in the selective encoding of duration, it is likely that attentional limits dictate the extent to which multiple durations can be effectively selected. In line with this idea, several studies have reported behavioral detriments when processing multiple, temporally overlapping durations^30–33^. Together with the current results, these findings point towards attentional limits in the encoding of duration, suggesting that duration processing is an effortful process with a limited capacity. Interestingly, capacity limits have been suggested to be related to the amount of overlap between the onsets and offsets of stimuli^30^. This suggests that attention for the entire interval might not be needed and that attending the on- and offset of an interval could be sufficient to encode its duration. It would be interesting to further explore this relation and investigate whether selectively attending the onsets and offsets of stimuli is sufficient to allow for the selective encoding of duration.

The extent to which attention can modulate responses to a certain feature have been shown to reflect complexity of the feature and its position in the visual processing hierarchy^6^. Several studies have shown that the magnitude of attentional modulation of after-effects increases as a function of the complexity of the encoded feature^4–9,34,35^. Furthermore, neurophysiological studies have demonstrated that attentional modulation of single cells responses to multiple stimuli increases as a function of the cells position in the visual processing hierarchy^6,12,13,36,37^. For example, the contribution of unattended features to a cells response have been reported to vary from ~30% in early visual area V2, to nearly 0% in cells in IT^12,37^. Since we found that the DAE reflected the attended duration and not the unattended duration (a 100% relative contribution of attended durations), our results support the notion that duration is a complex feature encoded by mechanisms located at higher-level areas of the processing hierarchy. This conclusion is in line with recent studies demonstrating low spatial selectivity of the duration after-effect^24,38^. This finding indicate that duration-selective neurons possess relatively large receptive fields, again suggesting a locus in higher-level areas of the processing hierarchy^24^.

In the current paradigm we manipulated attention by having participants focus their attention towards a specific location in space and processing duration information at that location. This design confounds attention towards a spatial location and attention towards the feature ‘duration’. As a result, it is not possible to conclude which of these types of attention is crucial for the attentional modulation observed in our experiments. A recent study investigating temporal recalibration found that attention towards the temporal order of events strongly modulated the resulting temporal recalibration compared to only attending the location at which the information is presented^39^. This result demonstrates that attention towards temporal features can be crucial for adaptive temporal processing; underscoring the possibility that the modulation observed here might rely on directing attention towards the feature ‘duration’. Future studies should aim to isolate specific types of attention to further understand its differential effects on temporal processing.

In our experiments we individually determined the duration of the auditory reference duration for each participant in an effort to create a reference duration that was perceptually equal to a 400 ms visual stimulus (a PSE of 400 ms). However, we observed an overall decrease in the perceived duration of the test stimulus in both Experiments (PSEs higher then 400 ms in all conditions), suggesting that the addition of the adaptation sequence influenced participants’ perception of our auditory/visual stimuli in an unexpected way. This is most clearly visible in Experiment 2 in the A4000|U400 condition, where no DAE is predicted but the average PSE was 472.1 ms (~18% compression). One possible explanation for this compression of perceived duration is that the shifts in spatial attention from the adaptation location to the test location caused compression of the test interval. Attentional shifts during (or close to) an interval have been shown to compress the perceived duration of that same interval^29^. Given the long delays between the offset of the final adapter (top-up) and the subsequent onset of the test stimulus (ISI - auditory reference stimulus - ISI), we did expect compression to occur in our design. However, since we do not know when during the delay participant shifted their attention, we cannot exclude that such duration compression effects occurred. Alternatively, it is possible that other visual adaptation after-effects confounded our measurement of the DAE. A recent study demonstrated localized duration compression following adaptation to the non-temporal features (i.e. orientation) of visual stimuli^32^. While we designed our experiments to reduce adaptation to non-temporal features by introducing spatial separation of adaptation and test stimuli, it is possible that some adaptation to non-temporal features occurred. This would lead to overall compression of duration at the test location, and could explain the overall duration compression we observed here. In our current experimental design it is not possible to demarcate the different possible source of the overall duration compression present in our results. Regardless of its origin, duration compression seems to occur across all conditions independently of our manipulation. As such, the observed compression does not affect our predictions and conclusion regarding the attentional modulation of the DAE observed in both experiments. Additional work will be needed to understand duration compression in these adaptation designs and improve similar designs for future studies.

In this study, an adaptation paradigm was used to probe the encoding of duration by measuring the effect of repeated encoding (or a lack thereof) on subsequent behavior. Although we find no evidence for a contribution of unattended durations to the DAE, the indirect measure used here does not allow us to claim that no encoding of the unattended duration occurred per se. For one, participants were aware of the unattended stimulus being presented, indicating that some information about the unattended stimulus was encoded. However, given the lack of a contribution of the unattended durations to the DAE, encoding of the unattended duration was either too sparse or too weak to lead to a measurable DAE. In other words, while we do not argue for the absolute lack of a representation of the unattended duration per se, we do argue that any information encoded about the unattended stimulus will not lead to a robust, stable representation, and as such will not impact the perception and behavior of the observer.

Our results demonstrate that attention gates the encoding of duration: attended durations lead to a DAE, while unattended durations do not. These findings are in line with earlier work suggesting attentional limits on the processing of duration and support the idea that duration encoding is an effortful process that requires gating by visual attention. We conclude that visual attention underlies the selection of relevant temporal information when multiple sources are present.

## Methods

### Participants

Data were collected from 12 participants (2 male, age M = 28, SD = 8.41) in Experiment 1, and 20 participants (7 male, age M = 25.55, SD = 7.72) in Experiment 2. All participants had normal or corrected-to-normal vision, and did not suffer from any neurological disorders. All participants were informed of their rights and gave written informed consent before the experiment started. Both experiments were approved by the local ethics committee of the Faculty of Social and Behavioral Sciences of Utrecht University and conducted in accordance with the guidelines expressed in the Declaration of Helsinki.

### Apparatus and stimuli

The same materials were used for both experiments. All visual stimuli were presented on a linearized Electron 22BlueIII CRT monitor (1280 × 1024, 100 Hz), controlled by a Dell OptiPlex 7040 workstation (Windows 10) using Matlab 2015b. For Experiment 1, gaze position measurements were taken using the EYE TRIBE tracker, sampling at 30 Hz. This eye tracker was controlled using the PyGaze software package and eye tribe toolbox for Matlab^40,41^. Furthermore, a chin- and headrest were used to increase head stability during tracking. Auditory stimuli consisted of a burst of white noise (60 dB, 0.01 ms ramp) presented through a Sennheiser on-ear headset.

For both experiments, all stimuli were presented on a gray background (9.6 cd/m^2^), accompanied by a white central fixation dot (64.7 cd/m^2^). Visual stimuli consisted of Gaussian blobs (40.8 cd/m^2^, 62% peak Michelson contrast, σ = 0.625°) presented at 5.8° of visual angle from fixation (Fig. 1). Adaptation stimuli were always presented left and right of fixation; test stimuli for the duration judgment task were presented below fixation. These distances and locations were selected so that participants could clearly distinguish each stream and the test stimuli. Additionally, we used this spatial setup to reduce adaptation to the non-temporal stimulus features of each stream^42^, while still assuring that adaptation to duration at both stream locations could be measured at the test location^24^.

### Procedure

Participants adapted to duration by viewing two streams of Gaussian blobs displayed to the left and right of a central fixation cross. Each stream consisted of repetitions of a single duration (ISI of 500–750 ms), with incidental duration oddballs (either shorter or longer) being presented on 10% of trials. To manipulate attention, participants were instructed to fixate the center of the screen and perform a duration-oddball detection task on the stream left or right of fixation. Performance on the oddball detection task was kept at 75% correct throughout the experiment by varying the oddball durations using an Accelerated Stochastic Approximation (ASA) staircase^43^. In Experiment 1, blobs in one stream lasted 200 ms while the blobs in the other stream lasted 800 ms. As a result, participants adapted in two conditions; attending the 200 ms stream (A200) or the 800 ms stream (A800). In Experiment 2, participants performed the oddball detection task in three conditions: repetitions of blobs lasting 200 and 400 ms, while performing the oddball task on the 200 ms blobs (A200|U400), repetitions of blobs lasting 200 and 400 ms, while performing the oddball task on the 400 ms blobs (A400|U200), or to repetitions of blobs that both lasted 400 ms, while performing the oddball task on one of the 400 ms streams (A400|U400). Adaptation to 400 ms repetitions was used here because it predicts no DAE when testing with a 400 ms reference duration. As such, the A400|U400 condition provides an appropriate baseline that is similar in visual presentation and task demands to the other conditions, while not predicting any DAE.

For all adaptation presentations, the longest duration was presented 100 times, while the number of repetitions for the shorter durations was set so that the two streams were approximately equal in total duration. For example, in Experiment 1, the 800 ms stimulus was presented 100 times, while the 200 ms stimulus was presented ~173 times. This method assured that presentation of the two streams terminated at around the same time, keeping the chance that the last stimulus belonged to either stream at ~50%. The order in which each stream of different durations was presented left or right of fixation was counterbalanced and presented in random order for each participant.

Following adaptation, we measured the DAE using a cross-modal duration judgment task. Each duration judgment trial started with top-up presentations of the oddball detection task with the aim of maintaining adaptation throughout the test period. In all cases, 4 repetitions of the longest duration were presented with the number of shorter durations selected to match the total stream duration for the longest duration stream. Following the top-ups, participants compared the duration of an auditory reference to that of a visual test stimulus and indicated which of the two durations they perceived as having a longer duration. For each trial, the duration of the visual test stimulus was varied using a Minimum Expected Entropy Staircase^44^.

In the duration judgment task the auditory reference always preceded the visual test stimulus, which should result in a time order error, with a longer perceived duration for the reference stimulus compared to the test stimulus^45^. To account for this error, we set the auditory reference duration to be perceptually equal to the 400 ms visual test stimulus for each individual participant at the start of the experiment. Participant completed a duration judgment task in which they compared a visual reference duration of 400 ms to that of an auditory test duration, the duration of which was varied using an ASA staircase^43^. Resulting estimates of the PSE were used to create the auditory reference used for the adaptation experiment. By doing so, we assured that the auditory reference duration in the adaptation experiment was perceived as being equal to the 400 ms (unadapted) visual test stimulus. The mean matched auditory reference durations for Experiment 1 and 2 were 362.37 ms (SD = 75.46) and 375.66 ms (SD = 102.90) respectively.

Each experimental session started with a practice block, in which participants practiced the duration judgments task (30 trials), followed by the auditory reference experiment (70 trials). Next, participants practiced the oddball detection task for each combination of attended durations and attended side. The aim of this practice session was to acquaint participants with the tasks and to derive initial discrimination thresholds, which were used to inform the adaptive staircase used to maintain detection performance at ~75% for the main experiment. For the main experiment, participants completed 4 blocks (50 trials each) in Experiment 1 and 6 blocks (30 trials each) in Experiment 2. This resulted in a total of 100 trials for each attention condition for Experiment 1 and 60 trials per attention condition in Experiment 2. The total sessions lasted ~3 hours for Experiment 1 and ~2 hours for Experiment 2.

### Gaze control

To assure that participants fixated the center of the screen and not the attended stimulus, gaze data was collected for 7 out of 12 participants in Experiment 1. Gaze position was tracked during adaptation and during duration judgments. What is more, duration judgments were contingent on accurate fixation, with deviations from fixation larger then 2.5° of visual angle leading to termination of the trial. Terminated trials were recycled.

We analyzed all successfully collected samples for both the adaptation (M = 97.24%, SD = 3.11%) and top-up presentations (M = 95.92%, SD = 3.81%). Analysis of these data showed that participants fixated within an area of 2° of visual angle on 90.66% (SD = 4.44%) of all successfully gathered samples during adaptation, and 91.91% (SD = 2.17%) of the samples collected during top-ups. Furthermore, participants failed to maintain fixation on 7.33% (SD = 3.8%) of all duration judgment trials. Together these results indicate that participants had little trouble following the instruction to maintain fixation at the center of the screen.

### Analysis

For both experiments we calculated the PSE for each of the attention conditions for each participant, by fitting a psychometric function using a logistic regression. These PSEs indicate the duration of the visual test stimulus that was perceived as being equal to the presented auditory reference. As such, higher PSEs indicate shorter perceived duration of the test stimulus and lower PSEs indicate longer perceived duration of the test stimulus. Average PSEs can be found in Figs 2 and 3 (see Supplementary materials S1 and S2 for individual data). The depicted error bars represent within-subject standard errors calculated using per-subject normalization of the data^46,47^. These standard errors reflect within-subject variability making them more informative of the outcome of the analyses of the within-subject effects reported here. The analyses for both experiments were conducted using Bayesian analysis in JASP^48,49^. For all analyses a common uninformative prior (Cauchy prior with width 0.707) was used.

In Bayesian analysis ‘significance’ is expressed in terms of a Bayes factor which indicates relative evidence between competing models (e.g. the model describing H_0_ vs. the model describing H_a_). For example, a Bayes factor: BF_10_ = 10 indicates that the collected data is 10 times more likely to occur under the H_a_ compared to the H_0_. Because the Bayesian framework allows for quantification of the evidence for both the H_a_ and H_0_ – something that is not possible using traditional inferential statistics – we can evaluate evidence that results in two conditions are not different from one another (as is applied in Experiment 2). To evaluate the evidence for or against each hypothesis we used a common rule of thumb in which BF_10_ > 3 is taken as sufficient evidence in favor of H_a_ and BF_10_ < 1/3 as evidence in favor of H_0_^50,51^. Importantly, it should be noted that while larger or smaller Bayes factors can be directly interpreted as larger amounts of evidence for their respective hypotheses, it does not inform about the effect size associated with the observed difference. To allow for insight into the effect size all reports will include the mean and standard deviation of each condition, and we encourage readers to evaluate the results visually in the provided figures.

### Data Availability

Experiment files, datasets, and analysis files for all experiments in this study are available in the OSF repository, 10.17605/OSF.IO/BE6U4.

## Electronic supplementary material

Supplementary materials
