# Supplementary material for: Attention Gates the Selective Encoding of Duration
Source: Sci Rep. 2018 Feb 6;8:2522. doi: 10.1038/s41598-018-20850-y (PMC5802729; doi:10.1038/s41598-018-20850-y)
Supplement: Supplementary file 1 — Supplementary materials [file 41598_2018_20850_MOESM1_ESM.pdf]

Attention Gates the Selective Encoding of Duration

Jim Maarseveen, Hinze Hogendoorn, Frans A. J. Verstraten, Chris L.E. Paffen

Supplementary Materials

Individual data

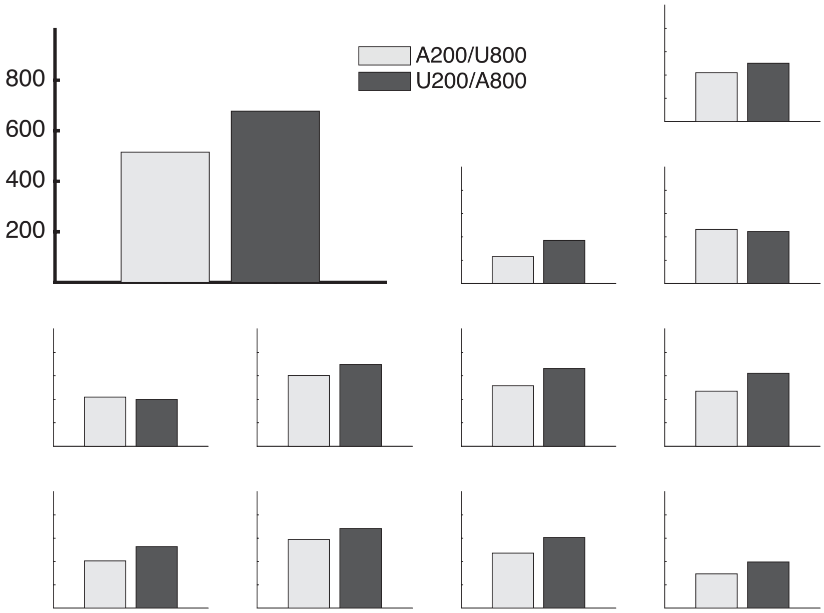

Figure S1. Individual data for Experiment 1, each graph represents median PSEs for a single participant. Higher PSEs reflect a shorter perceived duration for the test stimulus.

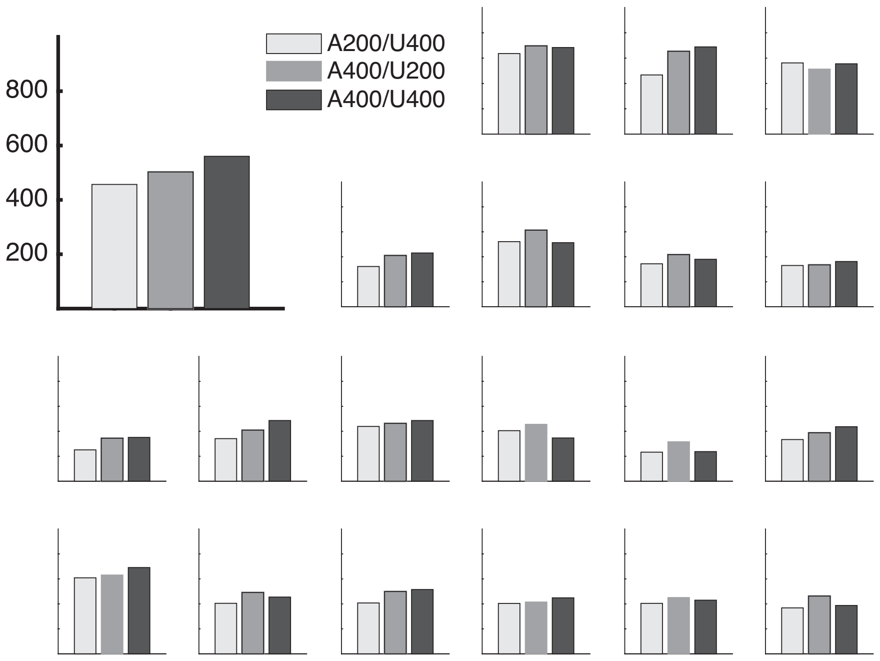

Figure S2. Individual data for Experiment 2, each image represents median PSEs for a single participant. Higher PSEs reflect a shorter perceived duration for the test stimulus.
